# Association of Bruxism with the Occurrence of Sleep Disorders and the Nature of Dreams

**DOI:** 10.3390/jcm14207269

**Published:** 2025-10-15

**Authors:** Sandra Szostak, Aleksandra Karykowska, Halina Kołodziej, Maciej Dobrzynski

**Affiliations:** 1 Department of Anthropology, Wrocław University of Environmental and Life Sciences, 5 Kożuchowska Street, 51-631 Wrocław, Polandhalina.kolodziej@upwr.edu.pl (H.K.); 2 Department of Pediatric Dentistry and Preclinical Dentistry, Wrocław Medical University, 26 Krakowska Street, 50-425 Wrocław, Poland; maciej.dobrzynski@umw.edu.pl

**Keywords:** insomnia, bruxism, teeth grinding, sleep, parasomnias, sleep disorders

## Abstract

**Background**: Bruxism is defined as repetitive activity of the masticatory muscles characterized by clenching or grinding of the teeth and/or stiffening or protrusion of the mandible, referred to as sleep bruxism or awake bruxism. It is believed that in generally healthy individuals, bruxism should not be considered a disorder, but rather a behavior that may be a risk factor (and/or protective factor) for certain clinical consequences. The aim of the study was to assess the occurrence and strength of the correlation between bruxism and sleep disorders (parasomnias and insomnia), as well as to evaluate the correlation between bruxism and the nature of dreams. **Methods**: The study involved 276 people aged 18–55. The research was conducted using an online survey in the period from January to March 2022. The author’s own questionnaire was composed of five segments of test questions (basic questions, bruxism severity test, Athens Insomnia Scale, parasomnia test, sleep evaluation test). The respondents were divided into groups based on the severity of bruxism, insomnia, parasomnias and the occurrence of dreams related to the oral cavity and teeth. Statistical analysis included Spearman’s correlation coefficients, chi-square test, Kruskal–Wallis ANOVA, and Mann–Whitney U test. **Results**: The analysis revealed a significant correlation between the severity of bruxism and insomnia symptoms. Participants with moderate and severe bruxism reported insomnia more frequently and with greater intensity. Similar correlations were observed with parasomnias and the frequency of dreams concerning the oral cavity and teeth. The strongest association with bruxism was found for physical dreams. Moreover, correlations were also noted between insomnia and parasomnias. **Conclusions**: These findings suggest that individuals experiencing bruxism, particularly sleep bruxism, may be more vulnerable to other sleep disturbances. Addressing one of these conditions could potentially have a beneficial impact on the others.

## 1. Introduction

Since 2013, bruxism has been defined as repetitive activity of the masticatory muscles characterized by clenching or grinding of the teeth and/or stiffening or protrusion of the mandible, referred to as sleep bruxism or awake bruxism. It is believed that in generally healthy individuals, bruxism should not be considered a disorder, but rather a behavior that may be a risk factor (and/or protective factor) for certain clinical consequences. Along with insomnia, these disorders are becoming an increasing problem affecting sleep quality and daytime functioning, which is reflected in deteriorating public health. Bruxism is one of the best-known disorders of the stomatognathic system [1,2]. However, the most recent consensus update in 2025 states that bruxism is considered a motor behavior rather than a disorder. Bruxism may serve as a sign of an underlying disorder or condition, such as sleep apnea, gastroesophageal reflux disease, or anxiety [3]. Moreover, bruxism can have negative clinical consequences in certain individuals. SB (sleep bruxism) can occur on its own or with other conditions, like OSA (Obstructive Sleep Apnea), gastroesophageal reflux, insomnia, headaches, pain in the mouth and face, periodic limb movements, rapid eye movement (REM) syndrome, and sleep epilepsy [4].

Symptoms of bruxism most often occur at night, but they can also appear during the day in stressful situations related to duties, family or work pressure [5]. While awake, bruxism is more common in women than in men. There are no significant gender differences during sleep [6]. Teeth grinding during the day affects about 23% of the general population, while teeth grinding during sleep occurs in approximately 21% of individuals globally. Overall prevalence (both types combined) is around 22.2%) [7]. The incidence increases significantly with age [8,9].

Sleep bruxism occurs predominantly in light NREM (non-rapid eye movement) sleep (stages 1 and 2) and during microarousals or transitions between sleep stages, with occasional occurrence in REM, i.e., patients presenting with neurological conditions, or comorbid sleep disturbances such as REM sleep behavior disorder and sleep apnea [10]. Therefore, it is included in the group of disorders consisting of the occurrence of abnormal or undesirable behavior during sleep, known as parasomnias. At the same time, it is the third most common parasomnia. It is strongly associated with the occurrence of night terrors and breathing disorders during sleep, which can lead to apnea [9]. The International Classification of Sleep Disorders (ICSD) provides diagnostic criteria for the diagnosis of bruxism, which is classified as a “sleep movement disorder” [11].

Treatment methods for this disorder include behavioral changes as well as pharmacological therapy. Therapeutic management involves the identification and subsequent elimination of the suspected cause and lifestyle factors that are contributing to the severity of symptoms. Minor anatomical malocclusions are considered predisposing factors as they frequently lead to the diagnosis of bruxism [12].

Since SB is included in the group of parasomnias, many researchers assume that the chance of its occurrence in subjects increases if they suffer from other sleep disorders from this group. In the REM phase, they may occur together with nightmares, especially characteristic of adults [13] Teeth dreams (TD) are often interpreted as nightmares, and more specifically, dreams about falling out or rotting teeth. They are one of the most common and universal dream themes. Moreover, they are particularly enigmatic, because they do not easily qualify for the “continuity hypothesis”, i.e., dreams about current and important experiences from waking life [14]).

### Aim of the Study

The aim of this cross-sectional study was to assess the occurrence and strength of associations between bruxism, sleep disorders (parasomnias and insomnia), and the nature of dreams.

## 2. Methods

The research material included 276 people aged 18–55 (242 women and 34 men) who were tested using the author’s own online questionnaire supplemented with publicly available forms for assessing the severity of sleep disorders. Participation in the study was voluntary and informed. Respondents completed consent forms to participate in the study. All participants had the right to withdraw at any stage of the study. The data collected was anonymized. The form was prepared specifically for the purposes of this study. The survey was divided into five segments, including sociodemographic questions, bruxism severity questions, Athens Insomnia Scale (AIS), parasomnia occurrence and severity form, and sleep pattern. The respondents were divided in terms of sex, bruxism diagnosed by the dentist (bruxism findings and its severity), prevalence of insomnia, prevalence of parasomnias, and frequency of dreams about the mouth and teeth (Table S1). The dental diagnosis of bruxism included a physical examination and a subjective assessment.

To assess the severity of bruxism symptoms, a test consisting of 11 questions was used to determine the frequency of occurrence of a given ailment. Questions about the severity of bruxism included pain in the temporomandibular joint, headaches, neck pain, teeth grinding, a feeling of clenched teeth, and tooth chipping. The questionnaire comprised 11 questions scored from 1 to 5, resulting in a maximum possible score of 55. Based on the quartiles calculated from the sum of the points obtained, the subjects were divided into four groups: advanced bruxism—over 18 points; moderate bruxism—13–18 points; light bruxism—8–12 points; no bruxism—less than 7 points.

In the analysis using AIS, the division proposed by Fornal-Pawłowska et al. (2011) was applied [15]. The survey questionnaire contained 21 questions concerning thirteen parasomnias. A five-point scale was used to assess their occurrence and severity. Each sleep disorder was assessed separately as well as together with others. In the assessment of sleep disorders, a total of 84 points could be achieved, which were then categorized based on tertile values: occurrence of parasomnias—5 points or less; sporadic parasomnias—6–14 points; frequent occurrence of parasomnias—more than 14 points.

Dreams were divided into two categories. Dreams related to oral problems (teeth falling out, eating hard vegetables or debris, and not being able to speak) could be recognized by three questions, with a maximum score of 12. The subjects were divided into groups according to the number of points obtained, where 0 points meant no such dreams, 1–2 points meant the appearance of these dreams occasionally, 3–4 points meant frequent dreams, and 5 points and above—very frequent dreams of this type. The most frequent type of sleep out of eight types of sleep was assigned a maximum of 4 points for each of them, where 4 points meant the occurrence of a given dream several times a week, while 0 points meant almost never.

### Statistical Analysis

The analysis was carried out using the statistical program Statistica 13.0. The sum of the points obtained for all respondents in each segment was calculated. Tests to check the normality of the distributions were performed. Considering that all distributions were significantly different from normal, the U-Mann–Whitney test was used to compare the median scores in the part of the questionnaire devoted to insomnia and parasomnias between the subjects with and without bruxism. The Kruskal–Wallis ANOVA test was used to assess the correlation between the occurrence of sleep disorders and the severity of bruxism. In cases where significant differences were found, multiple comparison tests of average ranks for all samples were performed in order to clarify which results were significantly different from each other.

The Chi-square test was used to assess the dependence of categorized groups of the severity of bruxism symptoms with the occurrence of insomnia, the frequency of parasomnias and the intensity of dreams focused on problems with the oral cavity and teeth. In order to investigate and determine the strength and direction of the correlation between the occurrence of bruxism and individual parasomnias and sleep characteristics checked in the questionnaire, Spearman’s correlation coefficients were calculated. The results for which the significance level was *p* ≤ 0.05 were considered statistically significant.

## 3. Results

There was no statistically significant difference in the intensity of insomnia symptoms between the subjects with bruxism and the subjects who did not show symptoms of this condition (Table S2). Despite this, individuals diagnosed with bruxism obtained a higher median score, indicating the borderline of the norm, while the remaining participants were in the range corresponding to undisturbed sleep.

A statistically significant association was observed between the severity of bruxism and the severity of insomnia (Table S3). The highest median scores were obtained by participants with advanced and moderate bruxism, and a statistically significant difference in insomnia intensity was found between individuals with advanced bruxism and those with mild bruxism (Table S4). The severity of bruxism symptoms was therefore significantly associated with the severity of insomnia (Table S5). Participants with moderate and severe bruxism more frequently reported symptoms indicative of probable insomnia, while those with mild bruxism or without bruxism most often scored within the range of normal, undisturbed sleep.

A statistically significant association was also found between bruxism and parasomnias. Participants with bruxism reported parasomnias more frequently (higher mean scores), whereas those without bruxism more often reported only sporadic parasomnia (Table S6). Moreover, the severity of bruxism symptoms was associated with the occurrence of parasomnias (Table S7). The highest median scores were observed in individuals with advanced bruxism, and the lowest in participants without bruxism. A significant difference in parasomnia intensity was found between individuals with advanced bruxism and those in other severity categories (Table S8). Similarly, participants with severe bruxism more frequently reported sleep problems categorized as parasomnias, while those with mild bruxism or without bruxism more often reported only rarely occurring disturbances (Table S9). Correlation analysis further confirmed a positive association between the severity of bruxism symptoms and the frequency of parasomnias (Table S10). Statistically significant associations were also found between bruxism and individual parasomnias. In addition, a positive association was observed between insomnia and parasomnias, particularly those that disrupt sleep continuity, such as night terrors, nightmares, sleep paralysis, exploding head syndrome, and somnambulism.

The severity of bruxism symptoms was further associated with the frequency of dreams about the oral cavity and teeth (Table S11). Participants with advanced bruxism significantly more often reported frequent or very frequent dreams of this type, while those without bruxism or with only mild symptoms more often reported either no such dreams or only sporadic ones. The strongest associations were observed between bruxism severity and physical dreams concerning somatic ailments experienced during the day, as well as with punishment dreams (Table S12).

It is important to note that the present results are based on cross-sectional data and demonstrate statistical associations, which should not be interpreted as causal relationships. Furthermore, even statistically significant correlations do not necessarily reflect clinical significance, and caution is warranted in extrapolating these findings to clinical practice.

## 4. Discussion

The study did not prove that people with bruxism were significantly more likely to report symptoms suggestive of insomnia. The average score of these subjects was in the range covering the borderline of the norm, indicating increasing problems with insomnia and being an indication to visit a specialist for a more accurate diagnosis. On the other hand, the subjects who were not diagnosed with bruxism had an average score in the range of undisturbed sleep. However, this discrepancy was not statistically significant, although a trend can be observed. Its alleged cause may be the sleep-disrupting nature of nocturnal bruxism, which, due to muscle activation, can cause pain in the head, neck and neck regions and contribute to mid-night awakening.

The obtained results are contradictory to many previous studies. One of them [16] proved the coexistence of insomnia and SB in middle-aged women (35–50 years). According to the assumptions of the researchers, insomnia is a very strong predictor of the occurrence of bruxism. Studies of Finnish shift workers also showed a statistically significant correlation between the discussed ailments [17]. The authors proved the influence of bruxism on the appearance of insomnia symptoms. A similar conclusion appears in the studies of Nachón-García et al. (2018) [18] and Hesselbacher et al. (2014) [19], indicating that people with bruxism are more likely to develop insomnia than people who do not have this disorder. However, the latest results obtained by Chattrattrai et al. (2022) [20] question whether the correlation between bruxism and insomnia is direct or results from other factors accompanying these disorders.

This hypothesis is supported by a recent study by Wieczorek et al. (2024) [21], which demonstrated that in individuals with severe sleep bruxism confirmed by polysomnography, anxiety and specific personality traits played a more decisive role than insomnia alone. The authors concluded that psychological vulnerability might be a key confounding factor in the bruxism–insomnia relationship. Similarly, a large network analysis conducted using data from the Netherlands Sleep Registry [22] showed that although sleep bruxism and insomnia often coexist, their direct association disappears when variables such as anxiety, stress levels, and emotional dysregulation are accounted for. These findings emphasize the importance of including psychological and environmental variables when evaluating the relationship between SB and sleep disturbances.

In our study, it was proved that there is a correlation between the intensity of bruxism symptoms and insomnia symptoms. A higher number of points scored in the segment of the questionnaire devoted to the intensity of bruxism was significantly associated with a higher result obtained on the Athens Insomnia Scale. This result may be due to the strengthening effect of individual ailments. More intense, strong teeth grinding leads to poor quality of sleep, while the problem with falling asleep or maintaining the continuity of sleep may contribute to stress, which intensifies bruxism. The obtained result is consistent with the results of research conducted among a Finnish cohort of twins born in 1975. It was confirmed that respondents who were diagnosed with bruxism reported sleepless nights depending on the severity of their bruxism. People who often struggle with SB were twice as likely to experience nightly insomnia symptoms compared to those who did not report this disorder. On the other hand, subjects with rare bruxism were 1.5 times more likely to experience insomnia compared to those without teeth grinding [23].

Further insight into this bidirectional relationship was provided by Blaszczyk et al. (2024), who analyzed patients suffering from comorbid insomnia and obstructive sleep apnea (COMISA) [24]. They demonstrated that in this group, bruxism episodes contributed to significant disruptions in sleep architecture, including reduced sleep efficiency and increased number of arousals. These findings support the idea that SB can aggravate insomnia symptoms by fragmenting sleep, particularly in individuals with coexisting sleep disorders.

Similar results were also obtained in studies by Itani et al. (2013) conducted on Japanese teenagers [25]. It has been proven that the more frequent occurrence of bruxism is associated with more frequent problems with falling asleep and maintaining sleep continuity. The study showed that bruxism was associated with the occurrence of parasomnias. Although sleep bruxism (SB) is no longer classified as a parasomnia, it frequently co-occurs with other arousal-related sleep disorders. Support for this result can be found in the study by Ohayon et al. (2001), which showed that parasomnias such as sleep paralysis, sleep hallucinations, and sleep talking were reported only in the teeth grinding study groups [9].

Other studies also correspond with the obtained result for children [26]. They prove, among others, that there is a significant correlation between the occurrence of SB in children and bedwetting. Researchers also showed that nearly 45% of children with bruxism spoke in their sleep, which was additionally confirmed by Seraj et al. (2010), who examined the association of bruxism with sleep talking and sleepwalking [27]. This is complemented by research conducted in a Brazilian dental clinic, which found that 64% of children with bruxism who come there talk in their sleep [28].

In this study, the severity of bruxism was related to the frequency of occurrence of parasomnias in each respondent. People with severe and moderate bruxism reported frequent occurrence of parasomnias, in contrast to those who did not have bruxism or suffered from a mild form. This may be due to the interplay of the intensifying ailments mentioned above. A more advanced form of bruxism may stimulate the occurrence of parasomnias more strongly, but this correlation may also be true in the reverse direction. This result is consistent with the results of Restrepo et al. (2017) [29] and Guo et al. (2017) [30], which showed that changes in the frequency of bruxism episodes are associated with a variable frequency of parasomnias, and more frequent teeth clenching is associated with more frequent sleep disorders. This result is also confirmed by the meta-analysis by Ribeiro-Lages et al. (2021) [14], which shows that the severity of bruxism is positively correlated with the appearance and intensity of parasomnias.

A positive correlation was also observed between the occurrence of insomnia and the occurrence of some of the examined parasomnias. A significant correlation was found between the severity of insomnia and the presence of parasomnias such as night terrors, nightmares, sleep paralysis, exploding head syndrome and somnambulism. A similar result and a possible explanation of the cooccurrence of these disorders can be found in the work of Ozgun et al. (2016) [31], which assessed the factors affecting the incidence of insomnia and parasomnia in children. The authors observed that many causes may affect the disorders. A small monthly family budget, low level of parental education, family problems and child allergies were associated with a higher incidence of both insomnia and parasomnias. The obtained result corresponds with the research by Bjorvatn et al. (2010) [32], which showed that subjects with insomnia more often complained of disorienting awakenings and nightmares.

In this study, the correlation between the intensity of bruxism and the frequency of dreams about the oral cavity and teeth was also proven. The result obtained can be explained in the Revonsuo (2000) study [33]. The authors concluded that dreams about teeth may be caused by dental stimulation, which is caused by a nocturnal episode of bruxism. This leads to the connection of the sensations evoked in the mouth with sleep. The hypothesis put forward by Revonsuo was examined by Rozen and Soffer-Dudek (2018) [34], confirming the correlation between dreams about teeth and the occurrence of bruxism. Researchers believe that the appearance of dental stimulation causes these types of dreams to occur. Yu’s research (2016) [35] confirms these results, finding the reasons for dreaming about dental problems in stimuli originating from an uncontrolled episode of bruxism during sleep.

The study observed a clear correlation between bruxism and the most common types of sleep, which were physical dreams and punishment dreams. Such a correlation between bruxism and physical dreams may result from the nature of the discussed type of dreams, because they consist in transferring real ailments into the realm of dreams. This would mean that the dominant physical sensation during these dreams in respondents is ailments involving the teeth, nearby muscles and the temporomandibular joint. The confirmation of such a hypothesis can be found in the study by Zadra et al. (1998) [36], which analyzed the cases of people who complained in their dream diaries about experiencing pain in the same places in the body both while awake and during sleep. Similar results appear in the work of Nielsen et al. (1993) [37], and the conclusions drawn by the researchers indicate that the pain appearing in dreams is a transfer of real sensations caused by the influence of an unspecified factor. In addition, pain in dreams may be associated with strong emotions experienced by the respondent and prompts them to act in the dream in a way that may provide some relief. Confirmation of these results can also be found in a study analyzing over 1600 entries from dream diaries [38]. The obtained results indicated that participants who struggled with pain ailments while awake significantly more often had dreams about pain in a specific body part. These findings highlight the complex and multidimensional nature of the relationship between bruxism, insomnia, parasomnias, and dream content. The results suggest that the coexistence and mutual reinforcement of these phenomena may contribute to significant disturbances in sleep quality and overall well-being. Future research should further explore the underlying psychological and neurophysiological mechanisms, as well as potential therapeutic strategies aimed at alleviating one disorder to improve outcomes in the others.

### Limitations

This study has several limitations. First, it is based on self-reported data, which may not accurately reflect the objective prevalence of sleep bruxism and sleep disorders. Questionnaire-based assessments are prone to recall bias and may overestimate the frequency of bruxism compared with polysomnographic studies. Second, the sample was predominantly female and covered a wide age range, which may have influenced the observed relationships. Finally, the cross-sectional design precludes causal inference between the variables examined. Future studies using objective diagnostic tools and more balanced samples are warranted.

## 5. Conclusions

The present study demonstrated significant associations between the severity of bruxism, insomnia, parasomnias, and the nature of dreams. Although individuals with bruxism were not significantly more likely to report insomnia symptoms than those without this condition, a clear positive association was observed between the intensity of bruxism and insomnia severity. Bruxism was also significantly related to the frequency of parasomnias and to the occurrence of dreams involving oral sensations and physical discomfort, suggesting shared physiological or psychological mechanisms.

These findings support the concept that bruxism should be regarded not as an isolated disorder but as part of a broader spectrum of sleep-related and stress-related phenomena. The coexistence and mutual reinforcement of bruxism, insomnia, and parasomnias may contribute to poor sleep quality and reduced well-being. Future studies employing objective diagnostic methods, longitudinal designs, and psychological assessments are needed to clarify the directionality and underlying mechanisms of these relationships and to develop targeted multidisciplinary treatment approaches.

## Data Availability

The original contributions presented in this study are included in the article/Supplementary Material. Further inquiries can be directed to the corresponding author.

## Supplementary Materials

The following supporting information can be downloaded at: https://www.mdpi.com/article/10.3390/jcm14207269/s1, Table S1: Number by sex, dentist-diagnosed bruxism, prevalence and intensity of bruxism, prevalence of insomnia, frequency of parasomnias, and frequency of oral dreams; Table S2: Bruxism and insomnia; Table S3: Bruxism intensity and insomnia; Table S4: Comparison of median scores depending on the intensity of bruxism [multiple comparison of average ranks for all trials]; Table S5: Bruxism intensity and insomnia intensity; Table S6: Diagnosed bruxism and parasomnias; Table S7: Bruxism intensity and parasomnias occurrence; Table S8: Comparison of median scores depending on bruxism intensity [multiple comparison of mean ranks for all trials]; Table S9: Severity of bruxism and prevalence of parasomnias; Table S10: Correlation of occurrence of bruxism, insomnia and parasomnias; Table S11: The intensity of bruxism and the frequency of dreams focused on the problem with the teeth and mouth; Table S12: Correlation of the incidence of bruxism with the most common nature of sleep.
