# Supplementary material for: Association of Bruxism with the Occurrence of Sleep Disorders and the Nature of Dreams
Source: J Clin Med. 2025 Oct 15;14(20):7269. doi: 10.3390/jcm14207269 (PMC12565331; doi:10.3390/jcm14207269)
Supplement: Supplementary file 1 [file jcm-14-07269-s001.zip › jcm-3874112-supplementary.pdf]

Table S1. Number by sex, dentist-diagnosed bruxism, prevalence and intensity of bruxism, prevalence of insomnia, frequency of parasomnias, and frequency of oral dreams

Variables	Categories	N	%
Sex	Female	242	87.68
	Men	34	12.32
Dentist diagnosed bruxism	Yes	35	12.68
	No	241	87.31
Bruxism	Yes	209	75.72
	No	67	24.28
Bruxism intensity	No bruxism	67	24.28
	Light bruxism	75	27.17
	Moderate bruxism	67	24.28
	Advanced bruxism	67	24.28
Insomnia	Standard	171	61.96
	Borderline of the norm	53	19.21
	Insomnia probable	52	18.84
Parasomnias incidence	Rarely	99	35.87
	Sometimes	86	31.16
	Often	91	32.97
Dreams about mouth and teeth problems	No	155	56.16
	Rarely	81	29.35
	Sometimes	28	10.14
	Very often	12	4.36

Table S2. Bruxism and insomnia

Diagnosed bruxism				Difference Y-N	
Yes (N=209)		No (N=67)			
Me	$\bar{X}$	*Me*	$\bar{X}$	*U*	*p*
5.00	5.02	4.00	4.73	6464.00	ns

Table S3. Bruxism intensity and insomnia

Bruxism intensity	*Me*	$\bar{X}$
No bruxism	4.00	4.73
Light bruxism	4.00	4.13
Moderate bruxism	5.00	5.16
Advanced bruxism	5.00	5.87
Difference	$H = 14.12$, $p < 0.01$	

Table S4. Comparison of median scores depending on the intensity of bruxism [multiple comparison of average ranks for all trials]

Bruxism intensity	[1]<br>Me = 4.00	[2]<br>Me = 4.00	[3]<br>Me = 5.00	[4]<br>Me = 5.00
No bruxism [1]		ns	ns	ns
Light bruxism [2]	ns		ns	<0.01
Moderate bruxism [3]	ns	ns		ns
Advanced bruxism [4]	ns	<0.01	ns	

Ns – non significant

Table S5. Bruxism intensity and insomnia intensity

Bruxism intensity		Insomnia			Marginal sum
		Standard	Borderline of the norm	Insomnia probable	
No bruxism	Lo /<br>Lo-Le	33.01<br>5.99	21.12<br>-4.12	12.87<br>-1.87	67
Light bruxism	Lo /<br>Lo-Le	36.96<br>8.04	23.64<br>0.36	14.40<br>-8.40	75
Moderate bruxism	Lo /<br>Lo-Le	33.01<br>-4.01	21.12<br>2.88	12.87<br>1.13	67
Advanced bruxism	Lo /<br>Lo-Le	33.01<br>-10.01	21.12<br>0.88	12.87<br>9.13	67
Total		136	87	53	276

$\chi 2 = 19.36$, $p < 0.05$, Lo-/Lo-Le -observed / expected frequencies

Table S6. Diagnosed bruxism and parasomnias

Diagnosed bruxism				Difference T-N	
Yes (N=209)		No (N=67)			
Me	$\bar{X}$	*Me*	$\bar{X}$	*U*	*p*
9.00	14.45	5.00	8.63	5280.00	<0.01

Table S7. Bruxism intensity and parasomnias occurrence

Bruxism intensity	*Me*	$\bar{X}$
No bruxism	5.00	8.63
Light bruxism	7.00	10.11
Moderate bruxism	8.00	13.22
Advanced bruxism	15.00	20.54
Difference	$H = 26.48, p < 0.05$	

Tabela S8. Comparison of median scores depending on bruxism intensity [multiple comparison of mean ranks for all trials]

Bruxism intensity	[1]<br>Me = 5.00	[2]<br>Me = 7.00	[3]<br>Me = 8.00	[4]<br>Me = 15.00
No bruxism [1]		ns	ns	<0.001
Light bruxism [2]	ns		ns	<0.001
Moderate bruxism [3]	ns	ns		<0.001
Advanced bruxism [4]	<0.001	<0.001	<0.001	

Table S9. Severity of bruxism and prevalence of parasomnias

Bruxism intensity		Parasomnia incidence			Marginal sum
		Rarely	Sometimes	Often	
No bruxism	Lo /<br>Lo-Le	24.03<br>9.97	20.88<br>-3.88	22.09<br>-6.09	67
Light bruxism	Lo /<br>Lo-Le	26.90<br>2.10	23.37<br>3.63	24.73<br>-5.73	75
Moderate bruxism	Lo /<br>Lo-Le	24.03<br>1.97	20.88<br>0.12	22.09<br>-2.10	67
Advanced bruxism	Lo /<br>Lo-Le	24.03<br>-14.03	20.88<br>0.12	22.09<br>13.91	67
Total		99	86	91	276

$\chi2 = 25.89$, $p < 0.05$, Lo-/Lo-Le -observed / expected frequencies

Table S10. Correlation of occurrence of bruxism, insomnia and parasomnias

Parasomnia		Bruxism	Insomnia
Night fears	*r*	0.23	0.20
	p	0.000	0.001
Somnambulism	*r*	0.22	0.14
	p	0.000	0.020
Catatrenia	*r*	0.26	0.24
	p	0.000	0.000
Sexsomnia	*r*	0.16	0.10
	p	0.007	Ns
Nightmares	*r*	0.21	0.18
	p	0.000	0.002
Sleeping paralysis	*r*	0.24	0.21
	p	0.000	0.001

Disorienting awakenings	*r*	0.23	0.17
	p	0.000	0.005
REM behavior disorder	*r*	0.25	0.03
	p	0.000	ns
Exploding head syndrome	*r*	0.20	0.13
	p	0.001	0.027
Nocturnal eating disorder	*r*	0.12	0.06
	p	0.047	ns
Bedwetting	*r*	0.14	0.06
	p	0.017	ns
Dream hallucinations	*r*	0.20	0.13
	p	0.001	ns
Total Parasomnias	*r*	0.36	0.29
	p	0.000	0.000

$p < 0.05$, r-Spearman

Table S11. The intensity of bruxism and the frequency of dreams focused on the problem with the teeth and mouth

Bruxism intensity		Occurrence of dreams focused on the mouth and teeth				Marginal sum
		No	Sometimes	Often	Very often	
No bruxism	Lo / Lo-Le	37.63<br>6.37	19.66<br>-4.66	6.80<br>-2.91	2.91<br>1.20	67
Light bruxism	Lo / Lo-Le	42.12<br>-0.12	22.01<br>4.99	7.61<br>-2.26	3.26<br>-2.61	75
Moderate bruxism	Lo / Lo-Le	37.63<br>2.37	19.66<br>1.34	6.80<br>-1.91	2.91<br>-1.80	67
Advanced bruxism	Lo / Lo-Le	37.63<br>-8.63	19.66<br>-1.66	6.80<br>7.09	2.91<br>3.20	67
Total		155	81	28	12	276

$\chi 2 = 31.75$, $p < 0.05$, Lo-/Lo-Le -observed / expected frequencies

Table S12. Correlation of the incidence of bruxism with the most common nature of sleep

Type of sleep		Bruxism
Repetitive dreams	*r*	0.25
	p	0.000
Erotic dreams	*r*	0.12
	p	ns
Lucid dreaming	*r*	0.14
	p	0.024
Punishment dreams	*r*	0.34
	p	0.000
Prophecy dreams	*r*	0.26
	p	0.000
Physical dreams	*r*	0.45
	p	0.000
Dreaming	*r*	0.19
	p	0.007
Reacting dreams	*r*	0.24
	p	0.000

$p < 0.05$, r-Spearman
